# Scalpel debridement has minimal effects on painful plantar calluses in older people: a randomised trial

**DOI:** 10.1186/1757-1146-5-S1-O27

**Published:** 2012-04-10

**Authors:** Karl B Landorf, Adam Morrow, Martin J Spink, Chelsey L Nash, Anna Novak, Adam R Bird, Julia Potter, Hylton B Menz

**Affiliations:** 1 Department of Podiatry, Faculty of Health Sciences, La Trobe University, Bundoora, Victoria, 3086, Australia; 2 Musculoskeletal Research Centre, Faculty of Health Sciences, La Trobe University, Bundoora, Victoria, 3086, Australia; 3 Faculty of Health Sciences, University of Southampton, Southampton, SO17 1BJ, UK

## Background

Plantar calluses are often associated with increased plantar pressure and foot pain, which can have a detrimental impact on the mobility and independence of an older person. Scalpel debridement is a key management strategy for painful calluses; however the effectiveness of this treatment in older people has not been rigorously investigated. The aim of this randomised trial was to evaluate the effectiveness of scalpel debridement in reducing plantar pressure and pain associated with forefoot plantar calluses.

## Materials and methods

Eighty participants aged 65 years and older with painful forefoot plantar calluses were recruited. Participants were randomly allocated to one of two groups: (i) normal scalpel debridement or (ii) sham (control) scalpel debridement. Participants were followed for six weeks. Both participants and assessors were blinded to the intervention. The primary outcomes measured were the difference between groups in pain (100 mm VAS) and barefoot plantar pressure (MatScan^®^ System).

## Results

Both groups experienced large decreases in pain following intervention (up to a 41.9 mm decrease in pain on a VAS). A systematic, but small beneficial effect on pain was noted in favour of the normal scalpel debridement group immediately post-debridement to 4 weeks post debridement (from 6.0 to 7.2 mm ANCOVA adjusted mean difference between groups). These values, however, are unlikely to be clinically important to a patient and there were no statistically significant differences (*P*<0.05) at any of the primary endpoints (immediately, and at 1, 3 and 6 weeks post-debridement). There were no statistically significant differences in plantar pressure between the two groups at any time-points.

## Conclusions

The findings of this trial indicate that scalpel debridement of painful plantar calluses has minimal effect. While we found a systematic effect favouring scalpel debridement, the benefits were small and not statistically significant. There was no change in plantar pressure following scalpel debridement.

